# Correlation of Aqueous, Vitreous, and Serum Protein Levels in Patients With Retinal Diseases

**DOI:** 10.1167/tvst.12.11.9

**Published:** 2023-11-06

**Authors:** Sabine Wilson, Juliane Siebourg-Polster, Bjoern Titz, Zhiwen Jiang, Francois Bartolo, Vincent Lavergne, Javier Gayán, Justus G. Garweg, Sascha Fauser, Andreas Dieckmann

**Affiliations:** 1 Roche Pharma Research and Early Development, F. Hoffmann-La Roche Ltd., Basel, Switzerland; 2 EFOR-CVO et Soladis, Champagne-au-Mont-d'Or, France; 3 EFOR-CVO et Soladis, Basel, Switzerland; 4 Berner Augenklinik, Bern, Switzerland; 5 Department of Ophthalmology, Bern University Hospital, University of Bern, Bern, Switzerland

**Keywords:** aqueous humor, vitreous humor, biomarkers, proteomics, retina

## Abstract

**Purpose:**

To further establish aqueous humor (AH) as a clinically suitable source of protein biomarkers in retinal diseases by evaluating the correlation of a large panel of proteins between AH, vitreous humor (VH), and serum (SE).

**Methods:**

We enrolled 60 subjects (eyes) with various non-infectious retinal diseases. AH, VH, and SE proteins were analyzed using the Olink Target 96 platform (1196 protein assays in total). We compared these three matrices in terms of quantification overlap, principal component analysis, and correlation.

**Results:**

In the AH, VH, and SE samples, 841, 917, and 1133 proteins, respectively, were consistently quantified above the limit of detection in more than 30% of patients. AH and VH shared 812 of these proteins. AH and VH samples overlapped along principal component 1, but SE samples were distinct. We identified 490 proteins with significant (false discovery rate [FDR]-adjusted *P* < 0.05) and relevant correlations (correlation coefficient > 0.5) between AH and VH, compared to only 33 and 40 proteins for VH and SE and for AH and SE, respectively.

**Conclusions:**

Due to a close correlation between protein concentrations in the AH and VH and a clear difference from the SE, AH has the potential to serve as a substitute for VH and may hold significance in identifying protein biomarkers and novel targets related to retinal diseases.

**Translational Relevance:**

This study further supports AH as a clinically suitable source of protein biomarkers in retinal diseases. In addition, the identified AH and VH correlations can inform the selection of protein biomarker candidates in future translational research.

## Introduction

Understanding the mechanisms underlying ocular diseases is crucial for improving diagnosis, monitoring disease progression, and developing effective treatments. Biomarkers have emerged as valuable tools in ophthalmology, providing measurable indicators of disease processes and treatment responses.^1^ Although imaging is widely used in the study of retinal diseases,^2^^–^^5^ it is not suitable for detecting the molecular changes associated with ocular pathophysiology. By complementing imaging investigations, molecular factors (including proteins, lipids, metabolites, miRNAs, and extracellular DNA, for example) in aqueous humor (AH) and vitreous humor (VH) have the potential to offer insights into the pathophysiology of retinal disorders.

Because of its close proximity to the retina, the VH is thought to more closely reflect retinal changes than the AH. Previous studies have shown correlations between VH proteins and the presence and severity of a variety of retinal disorders.^6^^–^^12^ However, diagnostic or investigative vitreous sampling is not routinely performed due to the well-documented potential risk of severe vision-threatening complications arising from such procedures; thus, the collection of VH samples is usually only performed during vitreoretinal surgery.^13^ On the other hand, AH sampling via anterior chamber paracentesis is widely considered a well-established procedure with relatively low safety risks when performed by an experienced ophthalmologist.^14^^,^^15^ Although AH is separated from VH by the lens and other structures, several studies have demonstrated correlations between certain proteins in the AH and VH, suggesting that AH may reflect vitreous levels of these factors.^16^^–^^21^ However, there is also conflicting evidence suggesting that the protein levels in the AH may not accurately match those detected in the VH.^22^^,^^23^ Overall, the relationship between protein levels in AH and VH is complex and may vary depending on the specific protein being studied, thus warranting further investigation.

To further address this knowledge gap, we enrolled 60 subjects (eyes) with different retinal diseases and investigated correlations of a large panel of protein markers among AH, VH, and serum (SE). For this, we utilized the Olink Target 96 technology platform (Olink Proteomics, Uppsala, Sweden). This proteomics platform utilizes proximity extension assays to measure protein expression levels using targeted, antibody-based assays.^24^ With its high sensitivity and specificity, the Olink Target 96 technology allows for the simultaneous measurement of over 1000 proteins in small sample volumes, making it ideal for the analysis of limited AH samples.

By establishing the level of correlation between AH and VH proteins, our study aimed to demonstrate the potential of AH to serve as a representative and valuable protein biomarker matrix for studying retinal conditions and to improve our understanding of intraocular diseases.

## Methods

### Patients

Each patient in this retrospective study required vitreoretinal surgery for the treatment of a vitreoretinal disease which was performed by a single surgeon (JGG) at the Clinic for Vitreoretinal Diseases, Berner Augenklinik (Bern, Switzerland) between 2017 and 2020. Before surgery, all patients had provided informed consent for the retrospective use and analysis of their coded patient data and biological samples for research purposes. This study was approved by the Cantonal Ethics Commission of Bern (2021–00248) and performed in accordance with the tenets of the Declaration of Helsinki in its latest version.

### Patient Samples

AH, VH, and SE samples were included from 22 eyes of otherwise healthy individuals and 38 eyes of patients with diabetes, adding six different retinal disease groups:

1. Otherwise healthy patients (no known systemic or local disease or treatment) with idiopathic macular hole (MH) or epiretinal membrane (ERM) (Healthy-MH/ERM; *n* = 15).
2. Otherwise healthy patients (no known systemic or local disease or treatment) with rhegmatogenous retinal detachment (RRD) (Healthy-RRD; *n* = 7).
3. Patients with diabetes and idiopathic MH or ERM but no diabetic retinopathy (Diabetes-MH/ERM; *n* = 12).
4. Patients with diabetes and RRD but no diabetic retinopathy (Diabetes-RRD; *n* = 8).
5. Patients with diabetes and non-proliferative diabetic retinopathy (NPDR; *n* = 12, with five mild, four moderate, and three severe cases) with vitreomacular traction, epiretinal membranes, and secondary macular holes.
6. Patients with diabetes and proliferative diabetic retinopathy (PDR) with significant vitreal opacification or macular pathologies requiring vitreoretinal surgery, but without vitreous hemorrhage (*n* = 6).

### Sample Collection

Aqueous, vitreous, and serum samples were collected on the day of surgery. All patients underwent small-incision (23-gauge) transconjunctival vitrectomy, in phakic instances combined with phacoemulsification and capsular bag intraocular lens implantation. At the beginning of surgery (i.e., directly after paracentesis and placing the ports), a sample of aqueous humor (∼80–100 µL) drawn from the anterior chamber was collected using a sterile 30-gauge needle on a 1-mL syringe. Vitreous samples were collected during vitrectomy surgery. Before fluid infusion was turned on, a non-diluted sample of vitreous (∼300 µL) was obtained with a standard 23-gauge microcutter from the middle of the vitreal cavity. Serum samples were obtained following standard procedures. All samples were transferred to microtubes and stored in the biobank of Berner Augenklinik within 30 minutes at −80°C until analysis.

### Proteomic Measurement

AH, VH, and SE samples (25 µL each) were shipped on dry ice to Olink Proteomics and analyzed using the Olink Target 96 platform. A single measurement was performed for each sample. The following 13 panels were assessed by Olink: cardiometabolic panel, cell regulation panel, cardiovascular II panel, cardiovascular III panel, development panel, immune response panel, inflammation panel, metabolism panel, neurology panel, neuro exploratory panel, oncology II panel, oncology III panel, and organ damage panel. A full list of the proteins in the selected panels is available in Supplementary Table S1. Protein levels were measured on a relative scale and presented as the normalized protein expression (NPX), which is an arbitrary unit on a log2 scale.

### Statistical Analysis

The Olink Proteomics data were subjected to extensive quality control (detectability, plate effects, density, outliers, etc.). Olink provides a limit of detection (LOD) for each protein based on the background noise generated in negative controls (buffer run as a normal sample).^25^ Nonetheless, the Olink protein expression data reported NPX values even for measurements below the LOD. Of the initial 60 patients, two outlier patients were removed from the analysis due to high missing rates in the measurements of one matrix (due to technical issues); thus, their data were not usable for correlation analysis. Additional missing values due to technical issues were very few and did not impact the statistical analysis. Proteins with low abundance (less than 30% of samples with NPX > LOD) were excluded from the statistical analyses in a data filtering step.

Principal component analysis (PCA) of all protein expression values was performed for all samples in all three matrices simultaneously. Spearman correlations were employed to estimate the relationship between protein levels across matrices. Correlations were estimated across the entire dataset and included all eyes, as the disease groups were too small to estimate correlations within each disease group independently. Correlation coefficients and false discovery rate (FDR)-adjusted *P* values per comparison between matrices are reported. Gene-set enrichment analysis was performed using Spearman correlation coefficients as the gene-level statistic and considering three gene-set collections from the mSigDB database (C5-GO:BP, C2-REACTOME, and C2-KEGG).^26^

## Results

### Patient Enrollment

We enrolled 60 subjects (eyes) with diverse retinal diseases in this study in order to generate sufficiently diverse protein profiles (Table) and used Olink Target assays to quantify panels of proteins in AH, VH, and SE samples.

**Table.** Demographic and Clinical Characteristics of Investigated Patients

Characteristic	Enrollment Set (*n* = 60)	Analysis Set (*n* = 58^a^)
Sex, *n* (%)		
Female	28 (47)	27 (47)
Male	32 (53)	31 (53)
Age (y), median (IQR)	71 (64–76)	71 (63–75)
Group, *n* (%)		
Healthy-MH/ERM	15 (25)	15 (26)
Healthy-RRD	7 (12)	7 (12)
Diabetes-MH/ERM	12 (20)	11 (19)
Diabetes-RRD	8 (13)	7 (12)
NPDR	12 (20)	12 (21)
PDR	6 (10)	6 (10)

Shown are the characteristics for the enrollment set and the analysis set (after exclusion of two outlier subjects).

a Two patients were excluded due to technical issues (see text).

### Protein Quantification Across AH, VH, and SE

We used the Olink Target 96 platform to quantify 1196 protein assays in AH, VH, and SE. For this, each sample was analyzed in 13 different Olink Target 96 panels (92 proteins × 13 panels), resulting in the quantification of 1161 distinct proteins with 35 protein assays repeated across different panels. After quality control of the analytical results, two patients were excluded (one patient as a multivariate outlier for VH and one patient due to a missing analytical panel in AH), resulting in a final analysis set of 58 patients that were considered in the subsequent analyses (Table).

**Figure 1.**
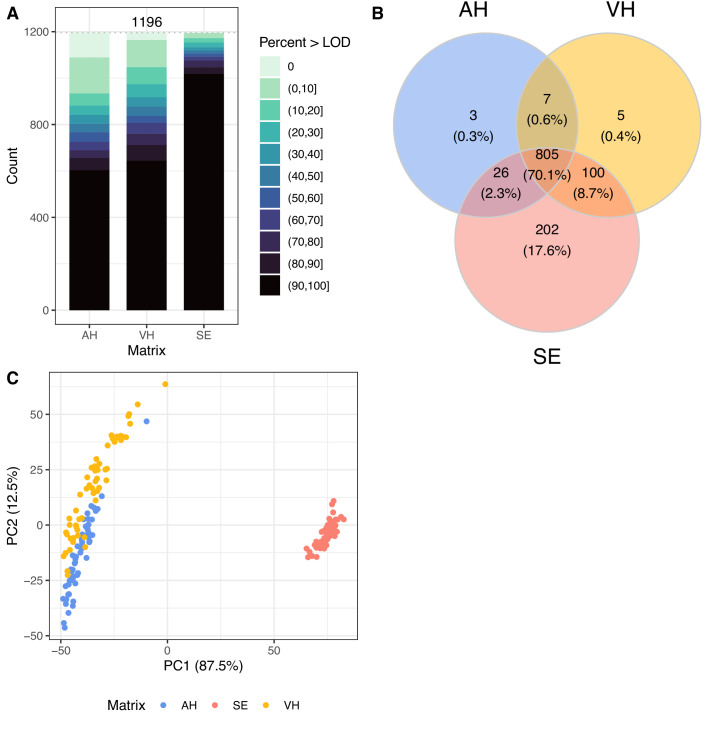
Quantification of proteins in AH, VH, and SE. (**A**) Number of proteins quantified above LOD for each matrix. The staggered bar plot shows the number of proteins quantified above the LOD across different percentage bins of the samples for each matrix. (**B**) Venn diagram illustrating the overlap of well-quantified proteins. Well-quantified proteins were defined as those quantified above the LOD in more than 30% of the samples of a given matrix. (**C**) Principal component score plot for principal components PC1 with 87.5% explained variance and PC2 with 12.5% explained variance. Each *point* represents a sample in this space with the matrix color-coded. The plot provides a visual representation of the relationship between samples based on their protein expression profiles, with PC1 separating SE from AH/VH and PC2 showing a lesser shift between the AH and SE samples.

We assessed the number of proteins quantified above the LOD for each matrix and found that SE had the highest number of proteins quantified above the LOD for the largest percentages of patients, whereas AH and VH showed lower and similar numbers (Fig. 1A).

To define well-quantified proteins for each matrix, we considered those that were most commonly quantified above the LOD using a threshold of more than 30%. We found that 1133 (94.7%), 917 (76.7%), and 841 (70.3%) of all proteins were well quantified in SE, VH, and AH samples, respectively. The Venn diagram in Figure 1B shows the overlap across these well-quantified proteins in the three matrices. We found that the majority of proteins (*n* = 805, 70.1% of target proteins) were well quantified in all three matrices, but SE had the highest number of unique proteins, and AH and VH also shared a large number of well-quantified proteins (*n* = 812). On these data, we performed PCA to visualize the relationships between the samples based on their protein expression profiles. The principal component score plot in Figure 1C shows that PC1 separates SE from AH/VH samples (87.5% of variance explained by PC1), whereas PC2 indicates a higher similarity between the AH and VH samples (12.5% of variance explained by PC1), demonstrating that a large number of proteins are well quantified and overlap in AH and VH, whereas protein profiles in SE are distinct.

### Protein Correlation Analysis Across AH, VH, and SE

**Figure 2.**
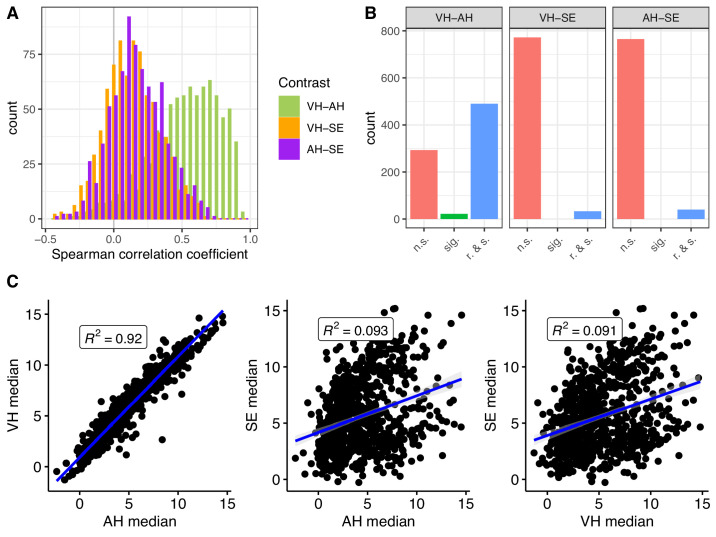
Protein correlation analysis across AH, VH, and SE. (**A**) Distribution of correlation coefficients between protein profiles across the three matrices. Spearman correlation was performed for proteins that were well quantified in all three matrices (i.e., proteins quantified above the LOD in more than 30% of the samples of a given matrix). (**B**) Numbers of significant and relevant protein correlations. Significant correlations were defined as those with a FDR-adjusted *P* < 0.05, and relevant correlations as those with a correlation coefficient > 0.5. n.s., non-significant; sig., significant; r. & s., relevant and significant. (**C**) Median protein value correlation analysis across the three matrices. Pairwise scatterplots were generated for each well-quantified protein, comparing the median protein values in the three matrices. *Blue lines* show the linear relationship (with 95% confidence intervals in *gray*), and Spearman *R*^2^ values are indicated.

Next, we investigated the correlation of proteins across AH, VH, and SE in the patient population under study. We focused on proteins that were well quantified in all three matrices and evaluated the distribution of Spearman correlation coefficients among proteins (Fig. 2A). Our results show that correlations between AH and VH were stronger than those between VH and SE and between AH and SE. Most VH–AH correlations were stronger than 0.3, spreading up to 1.0. The correlations between SE and the ocular fluids were generally modest, although most remained positive.

To summarize our findings, we determined the number of proteins with significant (FDR-adjusted *P* < 0.05) and relevant correlations (correlation coefficient > 0.5) (Fig. 2B). We found a significant and relevant correlation between AH and VH for 490 proteins, whereas only 33 and 40 proteins correlated significantly and relevantly for VH–SE and AH–SE, respectively (Supplementary Table S2). Furthermore, we compared the median NPX values in the three matrices for each well-quantified protein (Fig. 2C). Our analysis showed a high similarity between the median protein NPX values in VH and AH (*R*^2^ = 0.92), whereas there was only a weak correlation between VH and AH with SE (*R*^2^ < 0.1). It is worth noting that this analysis compares the relative ranking of median NPX values across proteins, whereas our previous analysis (Figs. 2A, 2B) compared how well the relative sample-to-sample differences are recapitulated across the matrices.

To assess potential variations in correlation strengths across distinct protein categories, we performed gene-set enrichment analyses using the recorded correlation coefficients (Supplementary Table S4). Notably, neither the VH–SE nor the AH–SE correlations revealed statistically significant gene-set enrichment. However, this analytical approach unveiled elevated VH–AH correlations within numerous gene sets related to immunity, cytokines, and chemokines.

Previously, when assessing the vitreous half-life of large molecules, a dependency on the molecular weight of the molecules was identified.^27^ We wondered whether the molecular weight also affected the correlation between protein levels in AH and VH. However, in the current study we observed only a slight negative trend with lower *R*^2^ coefficients for larger proteins with only 6.7% explained variance (*R*^2^) for AH versus VH (Supplementary Fig. S1).

In brief, a substantial number of proteins (∼41% of detectable proteins) are well correlated between AH and VH, whereas the correlation between these ocular matrices and SE is weak and limited to a few proteins.

### Selected Examples Relevant to Retinal Pathology

To further illustrate our findings, we have chosen representative protein examples from three important categories: angiogenesis regulators, immune-related proteins, and matrix metalloproteinases (MMPs). These categories were selected based on their crucial roles in ocular physiology and pathology.

**Figure 3.**
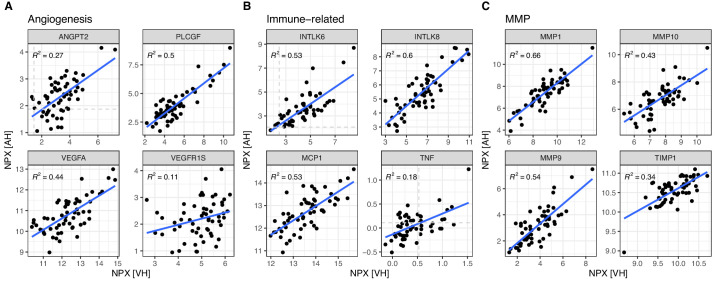
Selected examples relevant to ocular biology. Scatterplots of the protein quantities (NPX) in AH and VH. *Blue lines* show linear fit. Spearman correlation *R*^2^ values are indicated. (**A**) Angiogenesis regulators: ANGPT2 (ANG-2), PLCGF (PGF; PlGF), VEGFA, and VEGFR1S (FLT1). (**B**) Immune-related proteins: INTLK6 (IL-6), INTLK8 (IL-8), MCP-1 (CCL2), and TNF (TNF-alpha). (**C**) Matrix metalloproteinases/MMPs: MMP1, MMP10, MMP9, and TIMP1.

In Figure 3, scatterplots are presented to depict the protein expression levels of the selected proteins in AH and VH. Each scatterplot illustrates the correlation between AH and VH protein quantities, providing insights into their relationship within the ocular microenvironment.

Among the angiogenesis-related proteins (Fig. 3A), vascular endothelial growth factor A (VEGFA), a key target of anti-VEGF therapies, exhibited a strong correlation between VH and AH (*R*^2^ = 0.44). Placental growth factor (PLCGF) also showed a strong correlation (*R*^2^ = 0.5). Despite being close to the LOD, Ang-2 (ANGPT2) demonstrated a reasonable correlation (*R*^2^ = 0.27), whereas the soluble form of VEGF receptor 1 (VEGFR1S) exhibited a lower correlation (*R*^2^ = 0.11). Among the commonly investigated immune-related proteins, interleukin (IL)-6 (INTLK6), IL-8 (INTLK8), and monocyte chemotactic protein 1 (MCP1) showed strong correlations (*R*^2^ ranging from 0.53 to 0.6). However, due to most measurements falling below the LOD, tumor necrosis factor alpha (TNFα) displayed a relatively low correlation in this study (*R*^2^ = 0.18). In the MMP-related group, MMP-1, MMP-9, and MMP-10 demonstrated robust correlations (*R*^2^ between 0.43 and 0.54), whereas TIMP metallopeptidase inhibitor 1 (TIMP1) exhibited a slightly lower but still reasonable correlation (*R*^2^ = 0.34). Overall, the selected scatterplots further illustrate that many relevant proteins (including current drug targets or potential pharmacodynamic biomarkers) exhibit strong correlations between AH and VH, suggesting that AH sampling can be a convenient and valuable alternative to VH sampling to assess these proteins in retinal pathologies.

## Discussion

Numerous studies have already reported compelling evidence linking VH molecular factors to the presence and progression of diverse retinal disorders.^28^^–^^34^ However, the collection of VH samples is usually only performed during vitreoretinal surgery due to the potential risks of severe vision-threatening complications. These risks limit the widespread adoption of VH as a biomarker source in clinical studies.^13^ Hence, our primary question and objective in the current study were to determine whether VH and AH proteins exhibit a sufficient correlation to qualify AH as a relevant and convenient substitute for VH biomarker analysis.

To robustly evaluate the correlation of proteins among the three matrices (AH, VH, and SE), we evaluated 60 eyes with diverse retinal diseases, ranging from individuals with macular hole or epiretinal membrane to those with non-proliferative or proliferative diabetic retinopathy (Table) using the Olink Target 96 platform. Compared to other multiplex, antibody-based protein assay platforms, the Olink Target 96 platform offers several advantages, including its high sensitivity, quantitative performance, and the multiplexed measurement of up to 92 proteins per panel while utilizing an extremely low volume of sample per panel (1 µL).^24^^,^^35^ This technology has recently been demonstrated to be suitable for the analysis of VH samples, as it shows a strong correlation with an electrochemiluminescent sandwich immunoassay platform (MSD Technology Platform; Meso Scale Diagnostics, Rockville, MD).^35^

We successfully quantified 1133, 917, and 841 proteins in over 30% of SE, VH, and AH samples, respectively. As expected, the largest number of well-quantified proteins was observed in SE, given that the employed assay was initially developed for plasma/SE samples. Nevertheless, the ability to quantify more than 800 proteins in both ocular compartments, covering a wide range of functional categories such as immune response, metabolism, and cardiovascular processes, highlights the capability of this technology to facilitate comprehensive molecular profiling of ocular conditions. Notably, 812 identical proteins were found in both AH and VH, indicating the potential to identify common effects and relationships between these two matrices.

Principal component and correlation analysis supported a clear similarity of the protein profiles in AH and VH, especially when compared with SE profiles—reflecting the well-known distinct nature of the ocular compartment.^36^ Thus, the current study aligns with and extends previous research on the correlation of AH and VH proteins. We identified 10 previous studies that directly investigated the correlation of protein levels between AH and VH. These studies varied in sample size, ranging from 11 to 98 patients, and encompassed diverse retinal disease categories, including macular hole, retinal detachment, (non-)proliferative diabetic retinopathy, and retinal vein occlusion. Among these 10 studies, eight generally supported a direct protein correlation between AH and VH,^17^^–^^19^^,^^21^^,^^37^^–^^40^ whereas two studies did not provide robust evidence for such a correlation.^22^^,^^23^ For a summary of the correlation coefficients and the significance of these studies, refer to Supplementary Table S3. The most frequently investigated analytes were VEGF and IL-6, each covered by seven studies. VEGF and IL-6 exhibited significant correlations between AH and VH in six and five studies, respectively. The median correlation coefficients across all studies were 0.67 for VEGF (similar to the correlation coefficient of 0.67 in our study) and 0.74 for IL-6 (similar to the correlation coefficient of 0.73 in our study). These findings suggest a strong agreement between our study and previous findings. More generally, gene-set enrichment analysis also supported an especially high correlation of immune- and inflammation-related proteins, including cytokines and chemokines within the analysis panel.

To our knowledge, in terms of both the number of targeted protein measurements and population size, the current study represents the most comprehensive examination to date of correlations among AH, VH, and SE proteins (notably cytokines, chemokines, growth factors, and matrix metalloproteinases—crucial players in retinal disease pathology). Considering the range of observed correlation coefficients, we anticipate that this dataset should guide the selection of relevant AH proteins for future studies (i.e., the greater the correlation between VH and AH for a specific protein, the more significant its potential as an AH biomarker becomes). One additional strength of this study is the high sensitivity of the Olink Target platform in detecting more than 70% of all targeted proteins in more than 30% of AH and VH samples.

Although we found a significant and relevant correlation between AH and VH for more than 60% of the detected proteins, there was a substantial number of proteins that did not show such a correlation, at least under the conditions described in this study. Maurice^41^ extensively investigated the movement of fluids within the eye of rabbits and demonstrated that molecules exit the vitreous into the anterior chamber via slow diffusion from the anterior VH. This process has also been observed in humans, creating a gradient from the vitreous to the AH, which facilitates the diffusion of certain factors. This gradient may be due to the rapid elimination or accelerated degradation of proteins in the anterior chamber.^42^^,^^43^ Based on these findings, we hypothesize that the protein correlations observed between VH and AH in our study are influenced by the distinct diffusion (∼concentration) gradients of these proteins, and we speculate that the absolute concentrations of the proteins showing a good VH–AH correlation are higher in the VH (e.g., due to higher expression by the retina) than in the AH, which is supported by the higher median NPX values of VH proteins relative to their counterparts in the AH (Supplementary Fig. S2).

The VH acts as a selective barrier, allowing only certain molecules to diffuse through based on electrostatic and hydrophobic interactions.^44^^–^^48^ These interactions, influenced by the properties of the proteins, can result in different VH–AH diffusion behaviors among the different proteins, potentially explaining why we observed a good correlation for many but not all proteins even if their concentrations were potentially higher in the VH than in the AH.

Protein size, as indicated by molecular weight, did not appear to significantly affect the correlation between protein levels in AH and VH. There was only a slight negative trend observed for larger proteins, with an explained variance (*R*^2^) of 6.7% for AH versus VH (Supplementary Fig. S1), but polar or hydrophilic properties were not evaluated.

Shimada et al.^49^ measured VEGF concentrations in different regions of the vitreous and demonstrated that a VEGF concentration gradient already exists in the vitreous, with higher concentrations in premacular vitreous than in mid-vitreous and peripheral cortical vitreous, suggesting diffusion from the macular region to the periphery and from the posterior to the anterior globe. These findings, as exemplified by VEGF, led us to consistently collect VH from the same mid-vitreous area of the eye, aiming to reduce variations in protein concentrations associated with VH sample collection. With this, it can be assumed that the relative differences across protein levels in VH are well represented in our data, thus enabling robust and generalizable VH–AH and VH–SE correlation analyses, as correlation analyses depend only on relative differences.

Our study had certain limitations. The sample size was small, with only 58 subjects evaluated. Although we recruited a diverse group of patients with a variety of retinal diseases to enable a robust assessment of protein correlations across the three matrices, the resulting small numbers in each group hindered further exploration of significant differences across the different disease groups. To address this, our future research efforts will focus on expanding the sample sets accordingly to allow for more detailed AH protein analyses across different retinal diseases. Another limitation is associated with the Olink Target 96 platform used for protein quantification. Although this platform supports robust (targeted) relative quantification across samples, it does not provide absolute quantification values. Consequently, our data do not enable direct comparisons of concentrations across proteins within our study and do not allow us to compare absolute protein concentrations with other studies (e.g., to address the question of whether certain proteins have a higher abundance in AH than in other body compartments). Finally, with the current focus on protein measurements, this study cannot inform about other important biomolecules as biomarker sources such as lipids, metabolites, miRNAs, or cell-free DNA.

In conclusion, a considerable number of proteins exhibit reliable quantification and correlation between AH and VH. This underscores the relevance of AH in unraveling underlying disease mechanisms and in discovering new biomarkers and targets for retinal disease. Finally, we expect that the dataset generated will serve as a valuable resource for studying retinal diseases in the future.

## Supplementary Material

Supplement 1

Supplement 2

Supplement 3

Supplement 4

Supplement 5
