# Supplementary material for: Correlation of Aqueous, Vitreous, and Serum Protein Levels in Patients With Retinal Diseases
Source: Transl Vis Sci Technol. 2023 Nov 6;12(11):9. doi: 10.1167/tvst.12.11.9 (PMC10629536; doi:10.1167/tvst.12.11.9)

Supplementary Material

Correlation of Aqueous, Vitreous, and Serum Protein Levels in Patients with Retinal Diseases

Sabine Wilson[1,*], Juliane Siebourg-Polster[1,*], Bjoern Titz[1,*], Zhiwen Jiang[1,*], Francois Bartolo[1,2], Vincent Lavergne[1,3], Javier Gayan[1], Justus G. Garweg[4], Sascha Fauser[1], Andreas Dieckmann[1,#]

[1] Roche Pharmaceutical Research and Early Development, Roche Innovation Center Basel, F. Hoffmann-La Roche Ltd., Basel, Switzerland.

[2] Efor - CVO - Soladis, RACING PARK, 19 Rue des Rosiéristes Bâtiment B, 69410 Champagne-au-Mont-d'Or, France

[3] Efor - CVO - Soladis, Grosspeteranlage 29, 4052 Basel, Switzerland

[4] Berner Augenklinik, Bern, Switzerland and Department of Ophthalmology, Bern University Hospital, University of Bern, Bern, Switzerland

[*] contributed equally

[#] Correspondence: Andreas Dieckmann, Roche Pharmaceutical Research and Early Development, Immunology, Infectious Diseases and Ophthalmology (I2O), Roche Innovation Center Basel, F. Hoffmann-La Roche Ltd., Basel, Switzerland. e-mail: andreas.dieckmann@roche.com

Supplementary Tables

Supplementary Table S1: Selected Olink protein panels [see separate XLSX file]

Supplementary Table S2: Correlation results [see separate XLSX file]

Supplementary Table S3: Comparison of AH-VH correlation studies [see separate XLSX file]

Supplementary Table S4: Gene-set enrichment analysis for correlation results [see separate XLSX file]

Supplementary Figures

Supplementary Figure S1: Effect of protein molecular weight on R2. Scatter plot comparing the Spearman-based R2 coefficients with the molecular weight of the proteins for each matric combination. The black line shows the linear fit with confidence ranges. The Spearman R2 for this association is indicated.

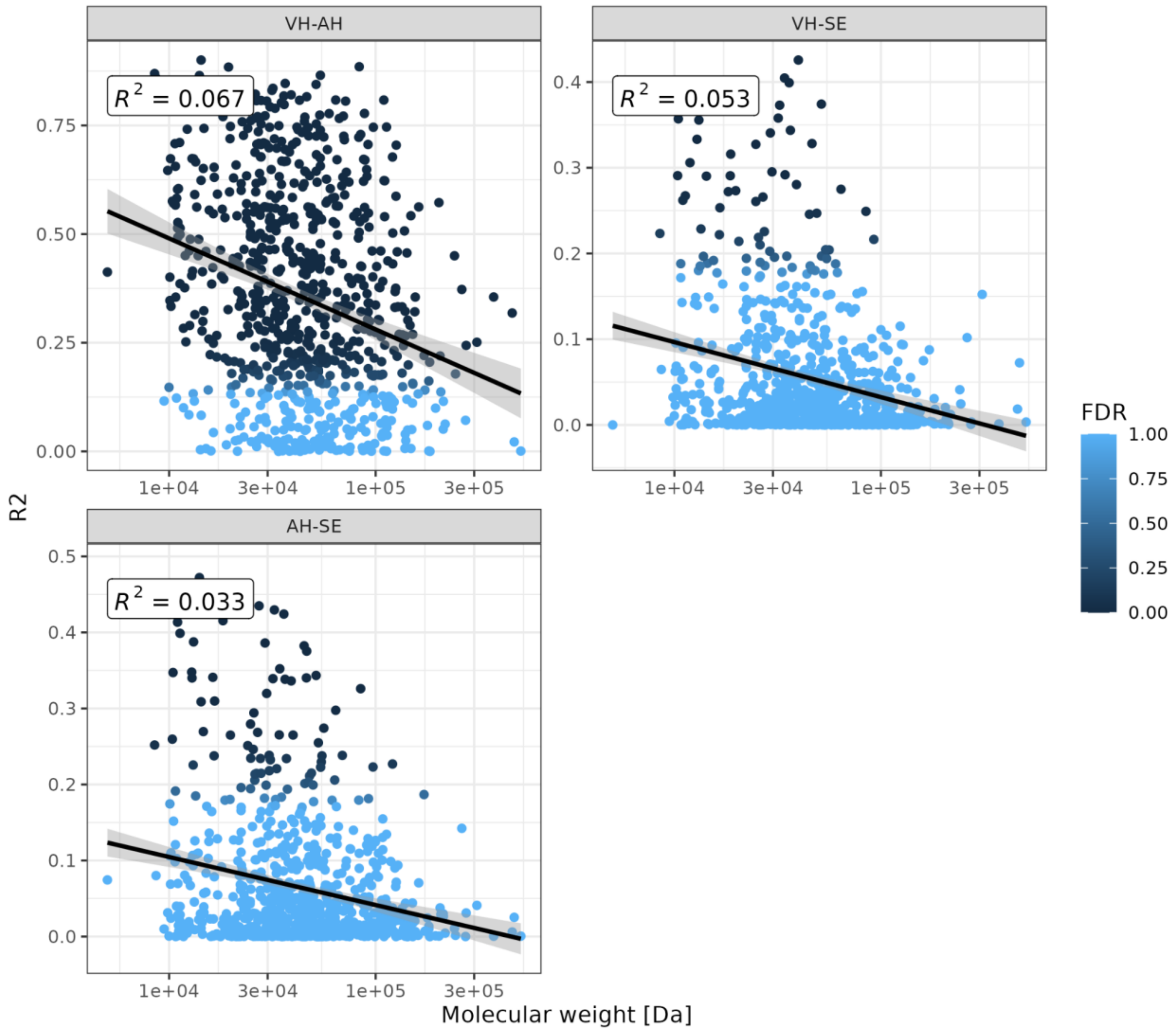

Supplementary Figure S2: Distribution plot comparing the median NPX values for proteins in VH and AH. The median is indicated by the red vertical line (median NPX value difference between VH and AH = 0.87).

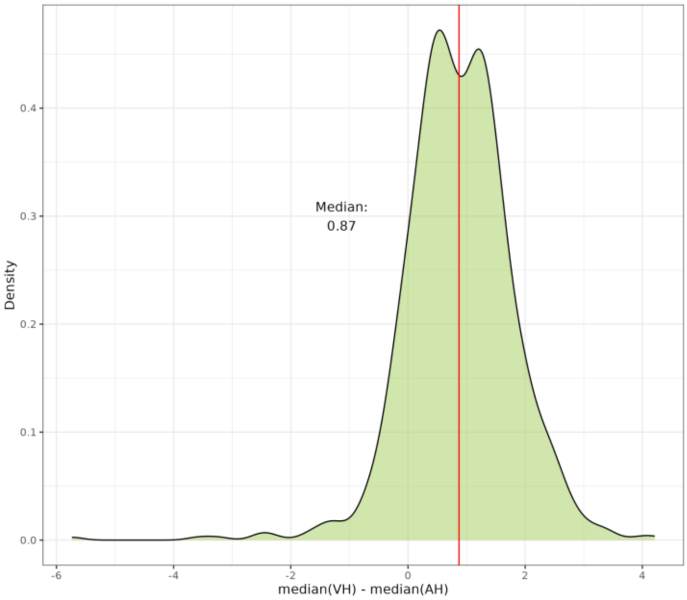

Supplement: Supplement 1 [file tvst-12-11-9_s001.pdf]
